# Sociodemographic determinants of depression, anxiety, and stress immediately after the COVID-19 pandemic: a cross-sectional study among university students in Saudi Arabia

**DOI:** 10.3389/fpubh.2023.1271536

**Published:** 2024-01-08

**Authors:** Ibrahim Alasqah, Abdullah Alhamdan, Mohammad Alhouri, Mohammed Alfehaid

**Affiliations:** ^1^Department of Public Health, College of Public Health and Health Informatics, Qassim University, Al Bukairiyah, Saudi Arabia; ^2^School of Health, University of New England, Armidale, NSW, Australia

**Keywords:** COVID-19 pandemic, mental health, anxiety, depression, stress, students, Saudi Arabia

## Abstract

**Background:**

The public health measures taken in educational institutions during the COVID-19 pandemic had complex influences on students’ mental health. This study aimed to evaluate the prevalence and sociodemographic determinants of Depression, Anxiety, and Stress immediately after the COVID-19 pandemic among students at Qassim University, Saudi Arabia.

**Methods:**

We conducted a cross-sectional study among Qassim University students in Saudi Arabia. All students registered for the academic year 2022 were invited to participate in a survey through social media. A total of 453 participants completed an online survey that included the DASS-21 for assessing the emotional states of Depression, Anxiety, and Stress..

**Results:**

The mean scores for Depression, Anxiety, and Stress, were 10.70, 11.18, and 10.40, respectively. At least 18% of the students reported experiencing moderate-to-severe levels of Depression, Anxiety, and Stress. The study showed that the feminine gender was associated with higher Depression, Anxiety, and Stress scores.

**Conclusion:**

Students in the current study described experiencing higher levels of Depression, Anxiety, and Stress during the COVID-19 pandemic. The findings of the present study can help universities take targeted measures to address the impact of a pandemic like COVID-19 on students’ mental health.

## 1 Introduction

The outbreak of coronavirus disease (COVID-19) has had a significant impact on the lives of people around the world, particularly after the declaration of a global pandemic by the World Health Organization on 11 March 2020 (1). In response to the pandemic, many countries have implemented a range of public health and social measures, such as travel and personal movement restrictions, shutting down of public areas, mandating face masks, and other measures to combat COVID-19. Saudi Arabia reacted quickly by imposing strict public health measures, including a travel ban, shutting down shopping centers and mosques, applying lockdown measures, implementing curfews, and suspending physical attendance to all educational institutions and workplaces except for critical jobs (2–4). The alarming increase in COVID-19 cases worldwide during the pandemic was associated with a considerable change in many aspects of people’s lives. This unprecedented situation impacted people’s psychological status. People were frightened and anxious. A recent systematic review reported that, during the pandemic, the prevalence of Stress was 29.6%, Anxiety was 31.9%, and Depression was 33.7% among the general population (5). Even before the COVID-19 pandemic, the world experienced various outbreaks over the past few decades, which caused post-traumatic Stress symptoms among people living in outbreak areas (6). Studies have also shown that the swine flu, Ebola, MERS, and SARS epidemics have caused mental health problems such as Depression, Anxiety, and fear of infection (7–9).

Universities and schools in Saudi Arabia have smoothly moved to virtual and distance learning during the pandemic. This shift was supported by the enhanced technology and digitalization of most services in the country (10). However, shifting the study mode and other social distancing measures have caused various challenges among students. Mental health issues, technological competency and accessibility, and the home learning environment were common challenges faced by Saudi Arabian students (11, 12).

The unprecedented experience of public health measures, particularly those related to teaching and learning, had complex influences on students’ mental health. The effect of COVID-19 on the mental health of university students during the pandemic has been investigated in many studies worldwide (13–22). A study conducted in six Middle Eastern countries reported that the total prevalence of Depression, Anxiety, and Stress was 57, 41, and 38%, respectively (23). A study conducted among rehabilitation students in Bangladesh revealed a high prevalence of Depression, Anxiety, and Stress symptoms during the COVID-19 pandemic (24). University students in France experienced similar psychological symptoms during the pandemic (25). In Saudi Arabia, many studies have investigated the impact of the COVID-19 pandemic on the mental health of university students. A systematic review found that the prevalence of Depression among medical students in Saudi Arabia ranged from 30.9 to 77.6%, with a mean prevalence of 51.5% (26). Another study conducted among medical and dental students in Saudi Arabia found higher levels of Depression (69.9%), Anxiety (66.4%), and Stress (70.9%) (27). According to a nationwide study in Saudi Arabia that aimed to investigate the psychological impact of COVID-19 among dental students, Depression, Anxiety, and Stress were highly prevalent during the pandemic (28). A study conducted among medical and non-medical students of Umm Al-Qura University, Saudi Arabia, reported that approximately 54, 53, and 38% of participants suffered from Depression, Anxiety, and Stress, respectively. The analyses showed that the two groups did not differ significantly in terms of Stress and Depression (29).

To the best of our knowledge, most of the published studies that assessed the psychological impact of COVID-19 among university students in Saudi Arabia were conducted among medical science students (26, 29–35). For example, a study was conducted to assess the prevalence of Depression, Anxiety, and Stress symptoms among medical students of Sulaiman Al Rajhi University, Al Bukayriyah, Saudi Arabia during quarantine and while learning online shortly after the announcement of documented COVID-19 cases in the Kingdom of Saudi Arabia (33). The experience of COVID-19 may differ between the medical and non-medical students. Studies conducted among all students mostly focused on the COVID-19 pandemic period (36–39). Therefore, from these studies, we do not know how the provenance changes post-pandemic when students start returning to their studies. For example, a study was conducted to assess the psychological condition of university students in Saudi Arabia during the COVID-19 pandemic. During the period between 4th and 18th of June 2020, students of Umm Al-Qura University in Saudi Arabia were invited to complete an online survey (39). This study aimed to evaluate the prevalence and sociodemographic determinants of Depression, Anxiety, and Stress during the COVID-19 pandemic among students at Qassim University, Saudi Arabia. Even if the pandemic is over, this finding will be relevant to preparedness for future pandemics. Moreover, the study will present disaggregated data by sociodemographic factors so that the universities can adopt targeted prevention strategies throughout Saudi Arabia.

## 2 Materials and methods

### 2.1 Study design and setting

We conducted a cross-sectional study to assess the psychological impact of the COVID-19 pandemic among students from all colleges within Qassim University, Saudi Arabia. Data were collected using an online questionnaire survey between 1 April 2022 and 31 May 2022. The survey was distributed via different channels, such as university email and social media. A total of 453 students across various colleges at Qassim University, Saudi Arabia have been included in the final analysis of this study.

### 2.2 Instrument

The Depression Anxiety Stress Scale-21 (DASS-21), a validated tool for screening to assess the psychological impact of the COVID-19 pandemic on mental health disorders among students, was administered (40). DASS-21 items were created to measure the emotional states of Depression, Anxiety, and Stress. The three DASS-21 scales have seven elements each and are further broken down into subscales. The Depression Scale assesses dysphoria, hopelessness, devaluation of life, self-deprecation, lack of interest/involvement, anhedonia, and inertia. The Anxiety Scale assesses situational anxiety, skeletal muscle effects, autonomic arousal, and subjective feelings of anxiety. The Stress Scale gauges issues with de-stressing, nervous arousal, and characteristics such as being easily agitated or angry, irritable/over-reactive, and impatient.

**Figure 1:**
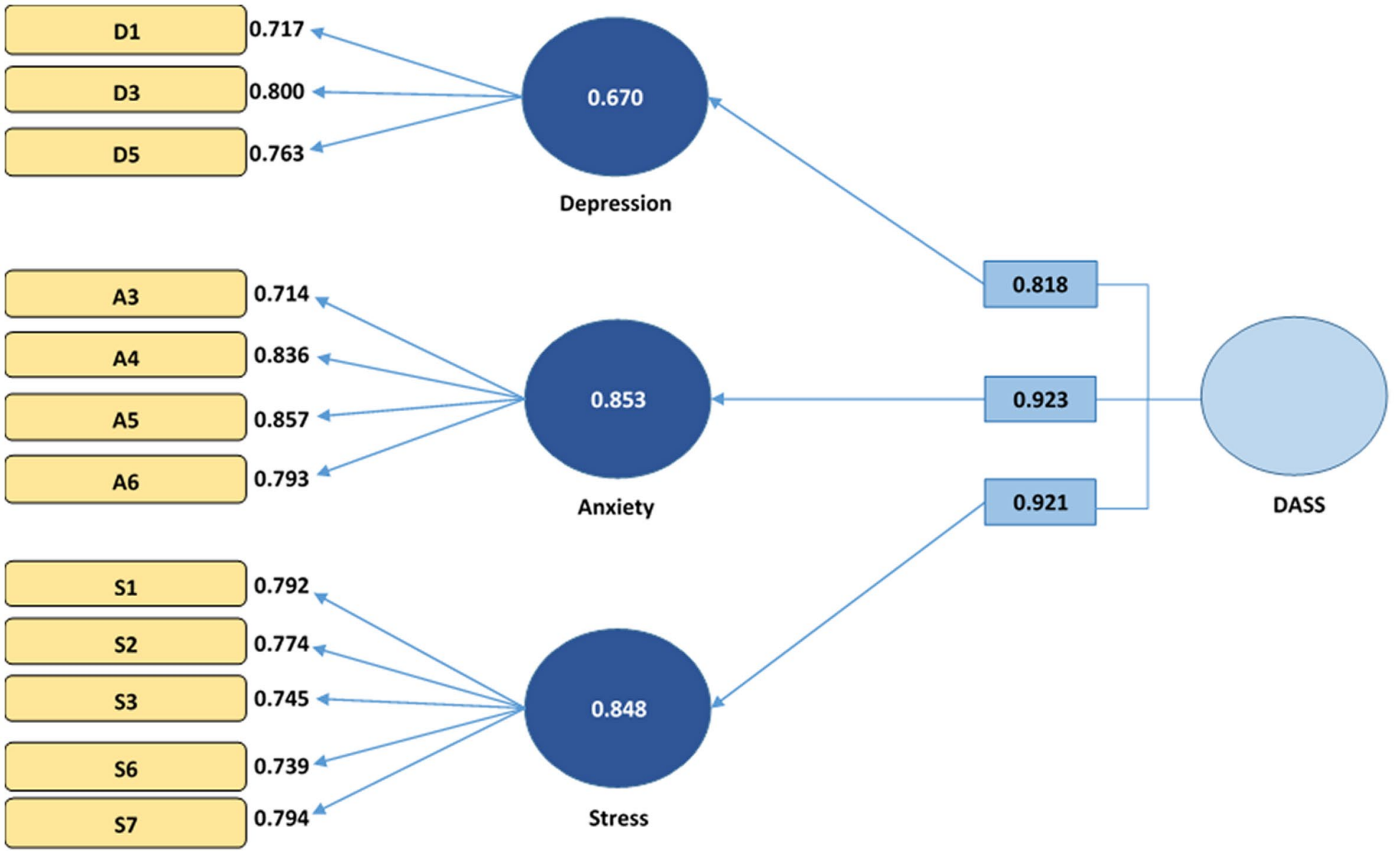
CFA (measurement model DASS).

The validity and reliability of DASS-21 were examined using confirmatory factor analysis. We used the following criteria to assess the measurement model given by Hair et al. (41): loadings >0.70, composite reliability (CR) >0.70, average variance extracted (AVE) > 0.50, VIF < 5, and Cronbach’s alpha >0.70. Henseler et al. (42) stated the value <0.835 for discriminant validity. From Tables 1, 2 and Figure 1, it is clear that DASS met the threshold, and the scale of DASS is reliable and valid.

**Table 1:** Measurement model (confirmatory factor analysis).

Variables	Items	Loading	CR	AVE	α	VIF
Depression	D1	0.717				1.224
	D3	0.800	0.805	0.579	0.637	1.294
	D5	0.763				1.241
Anxiety	A3	0.714				1.402
	A4	0.836	0.877	0.643	0.813	1.94
	A5	0.857				2.169
	A6	0.793				1.714
Stress	S1	0.792				1.801
	S2	0.774				1.667
	S3	0.745	0.879	0.591	0.827	1.640
	S6	0.739				1.691
	S7	0794				1.901

**Table 2:** Discriminant validity Fornell–Larcker.

Variables	Anxiety	DASS	Depression	Stress
Anxiety	0.802			
DASS	0.923	0.699		
Depression	0.687	0.818	0.761	
Stress	0.758	0.921	0.629	0.769

### 2.3 Procedures

The survey was announced through the communication channels of Qassim University and social media. An online questionnaire was used to collect data. The questionnaire was composed of four parts: I. The sociodemographic composition of students when COVID-19 was widespread; II. Student characteristics relating to the institution and education; III. Medical, psychiatric, and COVID-19 issues affecting students; IV. Students experienced Depression, Anxiety, and Stress Scale-21 (DASS-21) during the COVID-19 epidemic.

### 2.4 Variables

The dependent variables were Depression, Anxiety, and Stress, as measured using the DASS-21. The final score for the DASS-21 was determined by summing the results for the pertinent items and multiplying them by two. Scores for each subscale were divided into five levels: normal, mild, moderate, severe, and extremely severe. Stress was classified as normal (0–14), mild (15–18), moderate (19–25), severe (26–33), and extremely severe (34+). Depression was classified as normal (0–9), mild (10–13), moderate (14–20), severe (21–27), and extremely severe (28+). Anxiety was classified as normal (0–7), mild (8–9), moderate (10–14), severe (15–19), and extremely severe (20+).

### 2.5 Data analysis

After data collection, the information was updated, cleaned, coded, entered into an Excel file and then exported to Stata version 15 for analysis. Frequency and percentages were calculated for categorical variables and mean and standard deviation for continuous variables. Bivariate analysis was used to discover correlations between the dependent (Depression, Anxiety, and Stress) and independent variables using a *t*-test for binary independent variables and ANOVA for categorical independent variables. Then, multivariable analysis was performed using multiple linear regression to investigate the association between Depression, Anxiety, and Stress and sociodemographic variables. The findings of the study are then presented in the appropriate figures and tables.

### 2.6 Ethical consideration

Participants were informed that their answers would be confidential and used only for scientific purposes. The research was approved on 29 March 2022 by the research ethics committee of Qassim University, Saudi Arabia.

## 3 Results

### 3.1 Participant characteristics

A total of 453 students participated in this study. The average age of the participants ranged from 18 to 24 years, and most of the participants were male (*n* = 289; 63.8%). Fifth-year students comprised most participants (*n* = 101; 22.3%), followed by first-year students (*n* = 99; 21.9%). Furthermore, 96% of the participants reported living with their families. In addition, most (*n* = 311; 68.7%) of students attended a non-medical college (Table 3).

**Table 3:** Demographic and descriptive data of the students of Qassim University (*N* = 453).

Variable	n (%)
Age	
18–24 years	407 (89.8)
More than 24 years	46 (10.2)
Gender	
Men	289 (63.8)
Women	164 (36.2)
Academic year	
First	99 (21.9)
Second	85 (18.8)
Third	101 (22.3)
Fourth	92 (20.3)
Fifth	76 (16.8)
Living with	
Family	435 (96)
Alone	16 (3.5)
Accommodation with students	2 (0.4)
College type	
Medical college	142 (31.3)
Non-medical college	311 (68.7)
Received masks and hand sanitizer from the college	
Yes	290 (64)
No	163 (36)
Social distancing in place at the college	
Yes	229 (50.6)
No	224 (49.4)
Apologized or withdrew from the semester	
Yes	30 (6.6)
No	423 (93.4)
Have diagnosed with chronic medical illness	
Yes	38 (8.4)
No	415 (91.6)
Worried about their GPA scores and lack of knowledge	
Yes	337 (74.4)
No	116 (25.6)
Diagnosed with chronic diseases	
Yes	38 (8.4)
No	415 (91.6)
History of a chronic medical condition in their families	
Yes	211 (46.6)
No	242 (53.4)
Having a past psychiatric history	
Yes	46 (10.2)
No	407 (89.8)
Commitment to wearing a face mask	
Yes	363 (80.1)
No	90 (19.9)
Full implementation of COVID-19 protection protocol always	
Yes	305 (67.3)
No	148 (32.7)
Contact history with confirmed or suspected COVID-19	
Yes	292 (64.5)
No	161 (35.5)
History of relatives or acquaintances infected with COVID-19	
Yes	266 (58.7)
No	187 (41.3)
Fear of infected	
Yes	152 (33.6)
No	301 (66.4)

### 3.2 Institutional and educational variables during the COVID-19 pandemic

A total of 64% of students agreed that the college should supply sufficient masks and hand sanitizer. In addition, 50.6% of students agreed with a commitment to social distancing at a reasonable distance between students, faculty, and staff at the college. In addition, 93% of students apologized/withdrew from the semester, and 74% were worried about their GPA score and lack of knowledge (Table 3).

### 3.3 Medical illness, psychiatric, and COVID-19 complications

The majority (92%) of students reported not having any chronic medical illness, but 47% reported having a history of a chronic medical condition in their families. In addition, 90% of students reported not having a past psychiatric history, and 89% of students did not have a family psychiatric history. A total of 80% of students reported applying face masks regularly, and 67% agreed with the full implementation of the COVID-19 protection protocol. A total of 65% of students had a history of contact with confirmed or suspected COVID-19. In addition, 59% of students had relatives or acquaintances infected with COVID-19. One-third (34%) of students were afraid of infection (Table 3).

### 3.4 Description of Depression, Anxiety, and Stress

For Depression, Anxiety, and Stress, the mean scale scores were 10.40, 11.18, and 10.70, respectively. On the Stress scale, 70.42% of individuals reported normal levels of Stress, while 9.05% reported severe levels of Stress. However, 42.83% reported normal Anxiety, and 46.58% of the students had normal Depression levels (Table 4).

**Table 4:** Descriptive statistics of Stress, Anxiety, and Depression among Qassim University students (*N* = 453).

	Stress	Anxiety			Depression	
	*n*	%	*n*	%	*n*	%
Normal	319	70.42	194	42.83	211	46.58
Mild	41	9.05	30	6.62	82	18.1
Moderate	42	9.27	88	19.43	111	24.5
Severe	41	9.05	37	8.17	31	6.84
Extremely severe	10	2.21	104	22.96	18	3.97
Score, Mean (SD)	10.40	9.82	11.18	9.70	10.70	7.84

### 3.5 Sociodemographic variables associated with Depression, Anxiety, and Stress

The mean scores for Depression, Anxiety, and Stress symptoms among women were 13.56, 13.90, and 12.52, respectively. Men had mean values for Depression, Anxiety, and Stress of 9.66, 9.63, and 8.61, respectively. Male students had higher mean values than female students on Stress (*p* < 0.001), Anxiety (*p* < 0.001), and Depression (*p* < 0.001), respectively (Table 5).

**Table 5:** Factors associated with Stress, Anxiety, and Depression (Bivariate analysis, *N* = 453).

Risk factors	Stress			Anxiety			Depression		
	M	SD	*p*	M	SD	*p*	M	SD	*p*
Gender									
Men	8.6	9.2	<0.001	9.6	9.4	<0.001	9.6	7.9	<0.001
Women	13.6	10.1		13.9	9.6		12.5	7.4	
Age									
18–24 years	10.5	10	0.564	11.2	9.6	0.698	10.9	7.8	0.035
More than 24 years	9.6	8.6		10.6	10.3		8.4	7.4	
College type									
Medical	10.5	10	0.861	11.5	9.9	0.612	10.5	7.7	0.806
Non-medical	10.3	9.8		11.	9.6		10.7	7.9	
Academic year									
First	9.1	9.8	0.239	10.2	9.5	0.410	9.7	7.2	0.048
Second	10.8	9.6		11.8	9.9		11.6	8.1	
Third	12.1	10.4		12.5	9.4		11.2	7.3	
Fourth	10.3	9.9		10.8	9.3		11.9	9.1	
Fifth	9.5	9.1		10.3	10.5		8.9	7.2	
Living with									
Family	10.5	9.8	0.569	11.1	9.7	0.446	10.7	7.8	0.916
Alone or with students	9.1	9.6		12.9	10.1		10.9	8.4	

The study showed that the mean scores for Depression, Anxiety, and Stress levels for students aged 18–24 were 10.49, 11.24, and 10.96, respectively. The mean values for Stress, Anxiety, and Depression in students over 24 were 9.61, 10.65, and 8.39, respectively. Students aged more than 24 had higher mean values than those aged 18–24 years on Stress (*p* = 0.564), Anxiety (*p* = 0.698), and Depression (*p* = 0.035) (Table 5).

For medical students, the mean scores for Depression, Anxiety, and Stress were 10.56, 11.52, and 10.52, respectively. For non-medical students, the mean scores for Depression, Anxiety, and Stress were 10.76, 11.02, and 10.35, respectively. Stress scores for non-medical students were higher on average (*p* = 0.861). At the same time, the mean Anxiety and Depression scores were slightly higher among students from the medical colleges (*p* = 0.612 and *p* = 0.806, respectively) (Table 5).

The mean values of Depression, Anxiety, and Stress for first-year students were 9.68, 10.24, and 9.11, respectively; 11.62, 11.81, and 10.82 for second-year students, respectively; 11.21, 12.53, and 12.08 for third-year students, respectively; 11.89, 10.83, and 10.33, respectively, for the fourth-year students; and 8.87, 10.32, and 9.47, respectively, for fifth-year students (Table 5).

The multivariable analysis shows that gender (*p* ≤ 0.001) was associated with Stress, Anxiety, and Depression. Compared to first-year students, students studying in the third year experienced higher levels of stress (*p* = 0.005), anxiety (*p* = 0.027), and depression (*p* = 0.053). Similarly, compared to first-year students, students studying in the fourth year also experienced higher levels of depression (*p* = 0.006) (Table 6).

**Table 6:** Impact of demographic factors on Stress, Anxiety, and Depression (multivariable analysis).

	Stress				Anxiety				Depression			
Variable	Coef.	[95% Conf. Interval]		*P* > t	Coef.	[95% Conf. Interval]		*P* > t	Coef.	[95% Conf. Interval]		*P* > t
Gender												
Men	Ref				Ref				Ref			
Women	5.5	3.6	7.3	<0.001	4.8	2.9	6.7	<0.001	3.4	1.8	4.9	<0.001
Age												
18–24 years	Ref				Ref				Ref			
More than 24 years	−0.4	−3.8	3.0	0.819	−0.4	−3.7	3.0	0.831	−2.1	−4.8	0.7	0.136
College type												
Medical	Ref				Ref				Ref			
Non-medical	−1.0	−3.0	1.0	0.327	−1.4	−3.4	0.6	0.187	−0.4	−2.0	1.2	0.621
Academic year												
First	Ref				Ref				Ref			
Second	2.3	−0.5	5.1	0.107	2.0	−0.8	4.8	0.158	2.2	−0.1	4.4	0.060
Third	3.8	1.1	6.5	0.005	3.0	0.3	5.6	0.027	2.1	−0.0	4.3	0.053
Fourth	2.5	−0.3	5.3	0.077	1.7	−1.1	4.4	0.236	3.1	0.9	5.4	0.006
Fifth	1.0	−2.2	4.2	0.544	0.3	−2.9	3.5	0.862	0.3	−2.4	2.9	0.848
Living with												
Family	Ref				Ref				Ref			
Alone or with students	0.4	−4.3	5.1	0.869	3.3	−1.4	8.0	0.165	2.1	−1.6	5.9	0.267

## 4 Discussion

This study aimed to evaluate the prevalence and sociodemographic determinants of Depression, Anxiety, and Stress during the COVID-19 pandemic among students at Qassim University, Saudi Arabia. Our study found that at least 30% of the students experienced higher levels of Stress, 47% of students experienced higher levels of Anxiety, and 43% of students experienced higher levels of Depression.

During the pandemic, Qassim University ceased all operations, there were also several nationwide lockdown measures at the community level. In addition, students were afraid of being infected with COVID-19. Given the situation, it is natural that the students suffered from psychological distress. In our study, the mean levels of Depression, Anxiety, and Stress were 10.70, 11.18, and 10.40, respectively. These results were consistent with a study conducted in Saudi Arabia on the general population during the COVID-19 pandemic (43). A systematic review also found similar levels of abnormal Depression, Anxiety, and Stress among the general population (44). Furthermore, our findings are similar to a study conducted among nursing students from Hong Kong during the COVID-19 outbreak (45).

Our study found that, among Qassim University students, at least 30% of the students experienced higher levels of stress, 47% of students experienced higher levels of anxiety, and 43% of students experienced higher levels of depression. A study conducted among university students across Saudi Arabia during the pandemic (2 April 2020–5 April 2020) that used the DASS-21 tool also found similar levels of Depression (56%), Anxiety (40%), and Stress (41%). Another study conducted among university students in Riyadh found that 75% of the university students suffered different levels of depression during the pandemic (46). It is possible that the pressure students felt to complete their coursework successfully, earn top marks, and perform at their best during their education are contributing factors to these elevated levels of Depression, Anxiety, and Stress (47). Our study was conducted toward the end of the pandemic when the students started coming back to the university. Nonetheless, the prevalence of psychological symptoms suggests that students need professional support even after the pandemic is over, and the impact of the pandemic is present even after it is almost over. Due to a lack of data from the rest of Saudi Arabia, generalizations cannot be made about these findings. However, the data are comparable to previous studies conducted in Saudi Arabia. This suggests that university students in Saudi Arabia experienced abnormal levels of Depression, Anxiety, and Stress during and after the COVID-19 pandemic.

The Stress associated with attending university may be brought on by several things, including the need to memorize vast amounts of material, academic rivalry among students there, grade point averages, and a fear of failing (48). We evaluated the mental health of students in Saudi Arabia using the pre-validated DASS-21. It, therefore, had the additional benefit of making it simple to compare the findings with those of research reporting on variables related to mental health. This was especially true for most studies involving students because those studies used the DASS-21. The findings of the study were comparable to the research on Saudi Arabian dentistry students.

In our study, female students experienced more Depression, Anxiety, and Stress than male students. A systematic review, found that female medical students in Saudi Arabia also had a higher risk of depression than male students (26). In a study conducted among dental students in Saudi Arabia, depressive symptoms were higher in female students than in their male counterparts (47). Our findings were consistent with the research on the mental health of students in various other nations (49–53). Contrary to the results from our study, a study conducted among Jordanian university students found that male participants were influenced more than female participants (54). Clinical elements, including technical skills, were the most distressing for male students (54). However, the sociocultural context of Saudi Arabia may differ. This finding could be explained by the fact that, compared to men, women tend to have greater hyperactivity and cognitive disorders (55). Moreover, women experience more mental health problems due to the responsibilities they acquire from an early age, exposure to sexual abuse, and domestic violence (56).

In this study, the levels of Depression, Anxiety, and Stress did not differ according to the age of the student or field of study (Medical vs. Non-medical). A previous study conducted among Saudi Arabian university students did not find age and field of study to be associated with mental health symptoms (57).

According to the findings, first-year students experienced lower levels of Depression, Anxiety, and Stress than third-year senior students. A study conducted in Malaysia also found higher psychological symptoms among senior students (58). This could be due to the increasing workload in senior year and the fact that senior students are closer to graduation and must consider the future. A study conducted among orthodontic students before COVID-19 found no difference in Depression, Anxiety, and Stress levels among students from different academic years (59). This could be due to differences in the study context and sampling variations. It is also possible that COVID-19 may have contributed to higher stress among senior students in the middle of their university studies. According to a study by Naidu et al. on the causes of stress and psychological problems in college students, the general stress level increased as the students progressed (60). However, in our study, the experience of higher levels of stress was not consistent across all academic years. Further research is needed to understand this issue better.

This study has some important strengths. We used virtual data collection suitable for a pandemic, given that Saudi students are used to online activities. The data were collected immediately after the pandemic was almost over and when students started attending in-person classes. Therefore, our study findings represent the mental health status of students as they return to classes after the pandemic. We used the standard tool DASS-21, which allowed us to compare our findings with other studies. In addition, we have established the validity and reliability of DASS-21 for this study population. Finally, we have collected data from students of all disciplines, so the data are representative of all university students. Our study will contribute to the existing body of knowledge about the prevalence of stress, anxiety, and depression after a pandemic is over. Although we cannot conclude the impact of a pandemic from this study, it can highlight the need for public health measures to be taken post-pandemic. Moreover, our findings can shed light on the characteristics of students more prone to experiencing psychological symptoms due to a pandemic.

The study had some limitations. The non-probability nature of the sampling of the students may limit our findings’ generalizability. Given the fact that we had to reach the students virtually, it is possible that our sample is not representative of all types of students. Moreover, we only collected data from Qassim University students, so the findings can only be generalized to universities that are similar to Qassim University in terms of student profile. Nonetheless, our results are comparable to other studies conducted in Saudi Arabia among University students. We only collected data at a one-time point; thus, we cannot understand how psychological symptoms changed throughout the pandemic.

## 5 Conclusion

Students in the current study reported experiencing higher levels of Depression, Anxiety, and Stress immediately after the COVID-19 pandemic when they returned to classes. Depression, Anxiety, and Stress levels were associated with gender. These findings suggest that, even after the pandemic is almost over, students may experience psychological symptoms as they return to study. The findings of the current study can help universities gauge and treat current Stress, sadness, and Anxiety levels, as well as direct program development and mental health program implementation. Future research in this field should focus on developing and evaluating effective interventions to prevent and manage psychological symptoms among university students experiencing pandemics such as COVID-19.

## Data availability statement

The raw data supporting the conclusions of this article will be made available by the authors, without undue reservation.

## Ethics statement

The studies involving humans were approved by Committee of Research Ethics at Qassim University. The studies were conducted in accordance with the local legislation and institutional requirements. Written informed consent for participation was not required from the participants or the participants’ legal guardians/next of kin because virtual informed consent was obtained when the participants completed the online survey.

## Author contributions

IA: Conceptualization, Formal analysis, Funding acquisition, Investigation, Methodology, Project administration, Resources, Supervision, Validation, Writing – original draft, Writing – review & editing. AA: Conceptualization, Data curation, Formal analysis, Investigation, Methodology, Project administration, Software, Validation, Visualization, Writing – review & editing. MAlh: Conceptualization, Data curation, Formal analysis, Investigation, Methodology, Project administration, Software, Validation, Visualization, Writing – review & editing. MAlf: Conceptualization, Data curation, Formal analysis, Investigation, Methodology, Project administration, Software, Validation, Visualization, Writing – original draft, Writing – review & editing.
